# The Role of Emotional Regulation in the Relationship between Nurses’ Creative Style and Innovation Behaviors: A Cross-Sectional Study

**DOI:** 10.3390/nursrep13020071

**Published:** 2023-05-24

**Authors:** Ferdinando Toscano, Davide Giusino, Raffaello Diana, Tayebe Rahimi Pordanjani

**Affiliations:** 1 Department of Psychology, Alma Mater Studiorum—University of Bologna, 40126 Bologna, Italy; 2 Nephrology Unit, Rimini Hospital, Local Health Unit of Romagna, 47923 Rimini, Italy; 3 Department of Psychology, Faculty of Humanities, University of Bojnord, Bojnord 94531-55111, Iran

**Keywords:** nurses, creativity, innovation, emotion regulation, positive reappraisal, putting into perspective

## Abstract

Innovation is crucial to an effective healthcare system, and nurses are key figures in the innovation process. A potential factor behind innovation in nursing is the creative style of nurses. Creativity is an essential component of innovation. However, the relationship between creative style and innovation is complex and involves many different factors. Among them, given the nature of the nursing profession, we propose emotional regulation, or the ability to effectively manage one’s emotions. In this study, we hypothesize that two specific emotion-regulation strategies, positive reappraisal and putting into perspective, play a role in the relationship between nurses’ creative style and innovative behaviors. We tested a moderated mediation model using cross-sectional data from 187 nurses working in 3 university hospitals in Bojnord, Iran, in 2019. Our results show that positive reappraisal completely mediates the relationship between creative style and innovative behaviors, while putting into perspective moderates the relationship between positive reappraisal and innovative behaviors. These results suggest that nurses with a flair for creativity may be able to implement innovative behaviors in the workplace due to their ability to understand work-related situations and events positively. This may be especially true for nurses who can adopt alternative viewpoints. Our study discusses these findings by highlighting the importance of emotional regulation mechanisms in transforming nurses’ creativity into effective innovation. Finally, we provide suggestions for healthcare organizations to promote innovation as an added value in the healthcare environment and services provided.

## 1. Introduction

Innovation is a crucial driver of success in many organizations [1]. In hospitals and healthcare organizations, innovation corresponds to introducing new technologies, treatments, and approaches, improving patient care outcomes and patient satisfaction [2,3].

Nurses are key figures when it comes to introducing innovations in healthcare [4]. Due to their working at the front lines, nurses are often the best-positioned healthcare workers to identify areas for improvement in patient care. As such, nurses have the knowledge and expertise to suggest solutions, try out novel ideas and technologies, and provide feedback on their effectiveness. Hence, nurses play an essential role in driving innovation within the healthcare context, with the aim of improving the quality of patient care [2,4].

Innovation in nursing can help to improve the efficiency and effectiveness of the healthcare system by finding original ways to streamline processes and reduce costs [5]. Ultimately, this can benefit both patients and healthcare providers. For these reasons, it is relevant to understand which factors can influence innovation behaviors among nurses and how these can be managed and promoted.

Innovation arises in fertile ground. Previous research has demonstrated that organizations play a crucial role in fostering innovation within themselves, creating conditions, and providing support and resources available for innovation [6,7]. At the group level, leadership and communication strategies also contribute to innovation [8,9]. Nevertheless, innovation also depends on individual factors, related to the ability of individuals to think about things differently to how they have known or done them so far.

Creative style is one potential factor that can foster successful innovation processes within the healthcare context. It refers to the individual tendency to approach problems by generating new ideas [10]. Adopting a creative style can allow nurses to think out of the box and come up with unique and tailored solutions to problems they may encounter in their work practice, thus facilitating the provision of the best possible care. For instance, creative nurses can invent solutions to buffer the temporary shortage of supplies, such as adapting dressings for other purposes or, as happened in the early stages of the COVID-19 pandemic, using filters for an unintended purpose while waiting for the arrival of masks and suits suitable for the management of COVID-19 patients. Creative nurses can also develop new nursing techniques or practices, such as preventing and treating pressure ulcers, which new techniques are reducing over time [11,12]. Still, creative nurses can improve their experience, for example, by proposing revisions to shift management to aid their recovery, meeting the needs of ward staff, or even introducing stress-management strategies to involve the team. Hence, nurses adopting a creative style can draw on their knowledge and expertise to develop innovative approaches to care that can improve patient outcomes and enhance their overall experience [3,4].

Creative style might be expected to lead to a good deal of innovation in the workplace. Creativity is, indeed, a core component of innovation [13]. Innovation means coming up with new ideas and making them work well. This involves being willing to try new things and take risks, which is something people with a creative style do [10,14]. Thus, creative style might be considered an antecedent of innovation behaviors at work.

However, the relationship between creative style and innovation behaviors is not always straightforward. This is because such a relationship is a complex phenomenon involving several enabling factors [14].

Among others, emotion regulation might be an explanatory mechanism impacting nurses’ ability to innovate in their work [15,16]. Emotion regulation refers to the ability to manage one’s own emotions effectively [17,18]. This ability can help nurses remain focused when facing challenging situations and allow them to think and act more clearly when creative ideas have to be put into practice. By managing their emotion effectively, nurses might be more able to translate creativity into innovative solutions that can improve the quality of care they provide.

Despite the suggested importance of emotion regulation as a mechanism impacting nurses’ innovation behaviors, research so far has not provided evidence that supports or disproves the hypothetical role of emotion regulation in translating creativity into innovation among nurses. To fill this gap, in the present study, we investigated the mediating role of a major emotion-regulation strategy, positive reappraisal [19], in the relationship between nurses’ creative style and innovation behaviors. In addition, we explored the moderating role of another emotion-regulation strategy, putting into perspective [19], in the relationship between positive reappraisal and innovation behaviors.

The aim of the present study is threefold. First, we aim to advance the current understanding and the knowledge available about the relationship between creative style and innovation behaviors in the specific working population of nurses. Second, we aim to shed light on how emotion-regulation strategies, such as positive reappraisal and putting into perspective, can facilitate or hinder the process of turning novel and original ideas into actual and practical innovation, thus acting as underlying psychological mechanisms of innovation at work. Finally, we provide insights into managerial actions that might be deployed to enhance emotion regulation among nurses, with the broader objective of promoting innovation within healthcare organizations.

## 2. Hypotheses Development

The present study stems from two main theoretical propositions. First, we claim that positive reappraisal mediates the relationship between creative style and innovation behaviors. Second, we claim that putting into perspective plays a moderating role in the relationship between positive reappraisal and innovation behaviors. A moderated mediation was therefore proposed as well.

This section describes the main theoretical basis of our hypotheses.

### 2.1. Positive Reappraisal as a Mediator between Creative Style and Innovation Behaviors

In this study, we propose that the creative style of nurses can indirectly influence their adoption of innovation behavior at work through adopting the emotional regulation strategy of positive reappraisal. For this purpose, we define creative style as the individual’s unique way of generating and expressing ideas in a novel and original way [20], using imagination and intuition, and positive reappraisal as the emotional regulation strategy that involves reframing adverse events in a more positive light [21].

The creative style of nurses can directly influence their adoption of innovation behaviors at work, which are the actions and activities that individuals engage in to generate and implement novel ideas [14]. When nurses are faced with a new situation or problem, their creative style can help them to come up with novel and original ideas for addressing it. This can increase their willingness to try new things and take risks, which are essential for innovation [22]. For example, a nurse with a strong creative style may be more likely to suggest a new way of organizing the nursing team or to propose a novel approach to treating a particular medical condition.

However, creativity alone is not enough to promote innovation behavior. Research shows that nurses may be hesitant to try new things or take risks if they are overwhelmed by stress or negative emotions [23]. Furthermore, they may, for instance, face resistance from colleagues or superiors, or need more support and resources to implement innovations. In such cases, nurses may need to use emotional regulation strategies to cope with stress and maintain a positive outlook even under challenging circumstances.

Positive reappraisal is an emotional regulation strategy that involves reframing a stressful situation in a more positive and constructive way [24]. This may involve thinking of an advantage or a positive side of a negative situation, but it also may consist of reading the negative sides of the same phenomenon through perspectives not previously considered, which in any case modify the evaluation, even if still negative. For example, a nurse who faces resistance to an innovative idea can use positive reappraisal to see the situation as an opportunity to educate and persuade others or to learn from their feedback. This can help the nurse to keep a positive attitude, even in the face of obstacles and challenges. By using their creative style and emotional regulation strategies such as positive reappraisal, nurses can indirectly influence the adoption of innovation behavior at work. By generating novel ideas and solutions and coping with work situations in a positive way, nurses can overcome barriers and challenges and induce others to adopt their innovations.

For example, nurses with a strong positive-reappraisal tendency may be able to see a difficult patient as a chance to learn and grow, rather than as a source of stress. This can help nurses to cope with the situation in a more positive and constructive way. As a result, nurses may be more likely to experiment with new treatments or technologies and to seek out opportunities for professional development and growth. Hence, we hypothesize the following:

**H1.**  *Creative style is positively related to positive reappraisal.*

**H2.**  *Positive reappraisal is positively related to innovation behaviors at work.*

**H3.**  *Creative style is positively related to the adoption of innovation behaviors at work.*

### 2.2. Putting into Perspective as a Moderator between Positive Reappraisal and Innovation Behaviors

In the context of work, creative style and positive reappraisal may both contribute to innovation behaviors by providing individuals with the necessary skills and motivation to generate and pursue novel ideas. However, the relationship between positive reappraisal and innovation behavior may not always be straightforward. Thus, we extend the breadth of this relationship and deepen its explanation by proposing that the relationship between these variables is moderated by nurses’ putting into perspective, which refers to the ability to view events from multiple angles and to consider their context and relative importance [25].

In some cases, individuals may use positive reappraisal to reinforce their existing beliefs and attitudes, rather than challenging them. For example, nurses who are strongly attached to a particular way of doing things (e.g., treating a patient condition or applying a nursing procedure) may use positive reappraisal to justify their approach, rather than considering alternative approaches. This can hinder innovation behavior by limiting nurses’ willingness to try new things and take risks. This is where the moderating role of putting into perspective comes in. By taking a step back and considering the situation from multiple perspectives, that is, putting into perspective, nurses can challenge their existing beliefs and attitudes and consider alternative approaches to the problem. For example, they may be able to generate novel and original ideas for treating a patient condition or for organizing the nursing team in a more effective way. This can increase their willingness to try new things, take risks, and engage in innovation behavior [26].

For this reason, we believe that putting into perspective can enhance the relationship between positive reappraisal and innovation behavior. By enabling nurses to challenge their existing beliefs and attitudes and to consider alternative approaches to a problem, putting into perspective can promote innovation behavior by increasing individuals’ willingness to experiment. Hence, we posit the following:

**H4.**  *The relationship between positive reappraisal and innovation behaviors is moderated by putting into perspective, such that the positive influence of positive reappraisal on innovation behaviors is stronger when individuals can put a situation into perspective.*

**H5.**  *There is a positive indirect effect of creative style on innovation behaviors at work through positive reappraisal and putting into perspective.*

## 3. Materials and Methods

The presented study has a survey-based, cross-sectional, and correlational research design. In the following sections, information about participants, procedure, measures, and data analysis is summarized.

### 3.1. Participants

Two-hundred nurses working in twenty different wards at three teaching hospitals in the city of Bojnord, the capital of North Khorasan province of Iran, were involved in this study. There were no exclusion criteria. The sample is of convenience. After agreements with the hospitals’ management, the 20 nurse supervisors who had given their availability were contacted and encouraged to collect data in the department they coordinated, facilitating the contact with the nurses who had expressed their availability to participate in the study to their supervisors. Due to not having access to nurses who had freely withheld their consent to participate in the research, it was not possible for us to quantify the number of people participating in the population who were not involved in our study. For thirteen nurses, incomplete data were available. Thus, a sample of 187 nurses and the responses of 20 nurse supervisors in charge of coordinating ward nurses were considered for the study.

The sample of 187 nurses comprised 78.1% women and 21.9% men. The average age was 37.45 (SD = 7.61). The average tenure was 11.27 (SD = 8.10). To facilitate adherence to data collection, which took place in the sensitive context of a real workplace and in particular of health facilities, no information on hospital departments was collected.

### 3.2. Procedure and Measures

The study was conducted in accordance with the World Medical Association Declaration of Helsinki [27] and follows the STROBE (Strengthening the Reporting of Observational Studies in Epidemiology) guidelines [28], aimed at assuring comprehensive and transparent reporting of research methods in observational studies.

On three different days, after preliminary agreements with the organizations’ management, one of the researchers involved the nurses who were working at the three selected hospitals on duty at that time. The research questionnaires were completed with pencil and paper in nurses’ workplaces and private locations and then returned to the collecting researcher. The data collection occurred within one month in 2019, before the COVID-19 pandemic.

Participants were informed about the study’s objectives and the ethical standards of confidentiality and anonymity, made possible by anonymizing responses as soon as the collecting researcher matched responses between nurses and supervisors. Nurses did not have access to supervisors’ responses, and vice versa. Nurses were administered a questionnaire including three psychometric scales as follows.

Creative style was measured using an ad hoc Persian adaptation of the four self-report items from the Problem-Solving Style Questionnaire (PSSQ) by Cassidy and Burnside [20]. Responses were given in a 3-value forced-choice format, such as 0 = “False”, 0.5 = “I am unsure”, and 1 = “True”. A higher score indicated a higher level of creative style. An example item is “In my work, I think up as many ways as possible to handle the situation”. The construct validity of this measure has been demonstrated in previous studies [29], in which creative style appeared to be differentially predictive of different affective states and clinical disorders as compared to the other factors in problem-solving style, such as helplessness, problem-solving control, problem-solving confidence, avoidance style, and approach style.

Positive reappraisal and putting into perspective were measured using the Persian adaptation by Besharat and Bazzazian [30] of four self-report items from the Short Cognitive Emotional Regulation Questionnaire (CERQ-short) by Garnefski and Kraaij [31]. Two items were used to measure positive reappraisal, and other two items were used to measure putting into perspective. Responses were given on a 5-point Likert-type scale ranging from 1 = “Never” to 5 = “Always”. Higher scores indicated higher levels of positive reappraisal and putting into perspective. An example item for positive reappraisal is “I think I can learn something from the situation”, whereas an example item for putting into perspective is “I tell myself there are worse things in life”. The construct validity of this measure has been demonstrated in previous studies [21,31,32,33], in which positive reappraisal and putting into perspective showed differential relationships with different outcome measures, such as emotional problems and anxiety and depression symptoms, as compared to other cognitive emotional regulation strategies.

For supervisors’ evaluation of nurses’ innovative behaviors, information was collected from twenty supervisors on the same data-collection days as the nurses, after asking them to identify their supervisor. The very short questionnaire, one for each evaluated nurse, that supervisors completed used an ad hoc Persian adaptation of three items from the instrument developed by George and Zhou [34]. Responses were given on a 5-point Likert-type scale ranging from 1 = “Not at all characteristics” to 5 = “Very characteristic”. A higher score indicated a higher level of innovation behaviors. Each nurse was rated by one supervisor. An example item is “The nurse suggests new ways to achieve goals or objectives”. Contrarily to the others, we have no studies that have precisely demonstrated the validity of this 3-item scale as we used it, except for one study that also contained one of these items [35], in which the supervisors’ ratings of innovative behavior were correlated (r = 0.33, *p* < 0.001) with the objective measure of respondents’ disclosures (while controlling for their tenure). For this reason, to estimate the convergent validity, we report for this scale the Average Variance Extracted (AVE) value, which is for this study 0.58, well beyond its normally accepted cut-off (0.40 or 0.50).

The questionnaire also asked nurses for demographic information such as gender, age, job tenure, and level of education, to be used as control variables in the subsequently tested model. No demographic information was collected for supervisors.

### 3.3. Data Analysis

Data analysis was performed using the SPSS and AMOS software, both at version 26.

Confirmatory Factor Analysis (CFA) was conducted to assess the dimensionality of the administered questionnaire. We compared a one-factor model with a four-factor model, with each factor representing one of the main study variables, namely creative style, positive reappraisal, putting into perspective, and innovation behaviors.

Composite reliability (CR) and Cronbach’s alpha were computed to evaluate the reliability of the deployed psychometric measures. For CFA and CR, we adopted the cut-off values indicated by Hair and colleagues [36].

Before testing the model, we then computed descriptive statistics and correlations. Finally, we computed Model 14 from the PROCESS Macro for SPSS [37] to test the hypothesized moderated mediation model.

## 4. Results

This section reports the main findings subsequent to the statistical analyses performed in the present study.

### 4.1. Confirmatory Factor Analysis and Composite Reliability

CFA showed a better fit for the four-factor version (each item in its expected factor) than for the one-factor version (all items grouped in one factor) of the research model. The one-factor model showed the following fit indices: χ^2^ (44) = 398.74, χ^2^/degrees of freedom (df) = 9.06, Comparative Fit Index (CFI) = 0.53, Incremental Fit Index (IFI) = 0.54, Root Mean Square Error of Approximation (RMSEA) = 0.21, Standardized Root Mean Square Residual (SRMR) = 0.14. The four-factor model showed the following fit indices: χ^2^ (38) = 82.42, χ^2^/df = 2.87, CFI = 0.94, IFI = 0.94, RMSEA = 0.08, SRMR = 0.05. Thus, the dimensionality of the administered questionnaire was assessed favorably. Each item was loaded onto its respective factor, with all values being >0.40. CR showed above-threshold values for creative style (0.70), positive reappraisal (0.79), putting into perspective (0.87), and innovation behaviors (0.71). Thus, the goodness of fit and the structural validity and reliability of the model measures were supported.

### 4.2. Descriptive Statistics and Correlations

The results for the participants’ demographic data are illustrated in Table 1.

Descriptive statistics, Cronbach’s alphas, and Pearson correlations between study variables are shown in Table 2.

### 4.3. Model Testing

**Figure 1:**
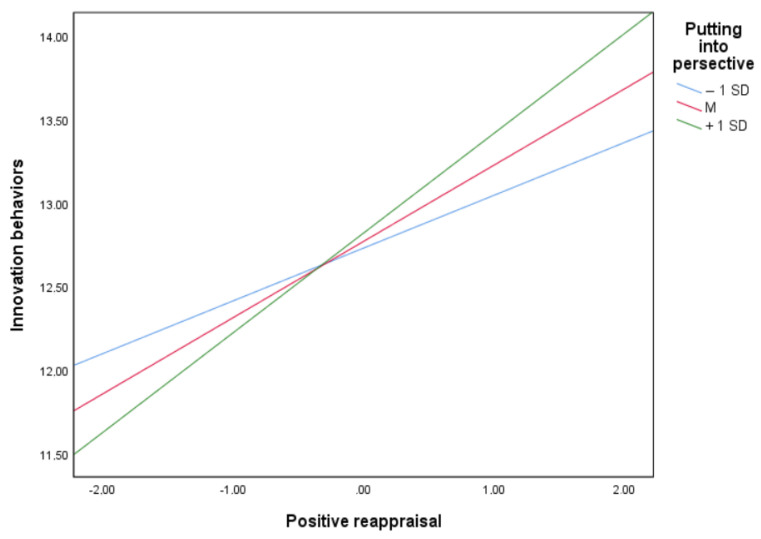
Moderation effect of putting into perspective in the relationship between positive reappraisal and innovation behaviors.

When testing the model, first, the empirical findings revealed creative style to be statistically significant and positively related to positive reappraisal (B = 0.55, *p* < 0.001). Thus, H1 was supported. Second, positive reappraisal was statistically significantly and positively related to innovation behaviors (B = 0.46, *p* < 0.001 when putting into perspective was considered at the average value; B = 0.32, *p* < 0.001 when it was at the −1 SD value; and B = 0.60, *p* < 0.001 when it was at the +1 SD value). Thus, H2 was supported. Contrary to expectations, creative style was not statistically significantly related to innovation behaviors (B = −0.08, *p* = 0.58). Thus, H3 was not supported. Control variables did not show any significant relationship, except for education level, which was positively related to both positive reappraisal (B = 0.48, *p* < 0.001) and innovation behaviors (B = 0.28, *p* = 0.04). Though the direct relationship between putting into perspective and innovation behaviors was not statistically significant (B = 0.02, *p* = 0.74), a statistically significant and positive interaction of putting into perspective was found in the relationship between positive reappraisal and innovation behaviors (B = 0.07, *p* = 0.02). Thus, H4 was supported, because the moderation of putting into perspective in the relationship between positive reappraisal and innovation behaviors was confirmed, as shown in Figure 1.

**Figure 2:**
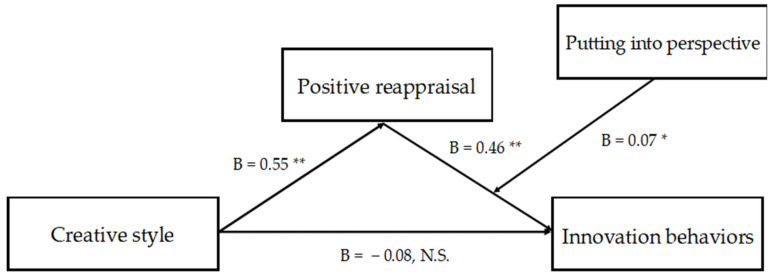
Results of the study model. * *p* < 0.05. ** *p* < 0.01.

Finally, analyzing the indirect effects of creative style on innovation behaviors, the results show that, considering average and ±1 SD values of putting into perspective, creative style and innovation behaviors were in all circumstances positively related, with the minimum effect (point estimate = 0.17 [0.05; 0.36]) at the −1 SD value of putting into perspective and the maximum effect revealed when putting into perspective was at its +1 SD value (point estimate = 0.33; [0.15; 0.57]). Hence, the results support H5, positing an indirect effect between creative style and innovation behaviors through positive reappraisal and putting into perspective. However, these indirect effects showed no differences when putting into perspective was considered at its different values, because the index of moderated mediation proved to be not significant (point estimate = 0.04; [0.00; 0.09]). The direct results of the model are represented in Figure 2. The conditional indirect effect is shown in Table 3.

## 5. Discussion

In the present study, we aimed to investigate the explanatory mechanisms of the relationship between creative style and innovation behaviors at work among nurses. In other words, we aimed to identify under which underlying circumstances or factors individual nurses’ tendencies to creativity actually produce innovative behaviors in the healthcare workplace context. We focused on the psychological variable of emotion regulation and, specifically, on the interacting role of two relevant emotion-regulation strategies: positive reappraisal and putting into perspective.

Our findings show a full mediation of positive reappraisal in the relationship between creative style and innovation behaviors of nurses. Furthermore, a moderating role of putting into perspective was highlighted in the relationship between positive reappraisal and innovation behaviors.

These results suggest that nurses with a subjective predisposition to act and think with creativity might be capable of implementing innovative behaviors in their workplace because they are also able to understand situations at work in a positive fashion, with potential effects on the tasks performed, their management, and patients. Furthermore, positively biased nurses might more easily introduce innovations in their work when they are psychologically flexible enough to see things from different or alternative angles and viewpoints. Overall, nurses with high positive reappraisal and putting into perspective might be better equipped to overcome challenges and barriers that may prevent creative ideas and input from translating into actual solutions, useful novelties, and impactful, original changes in the healthcare workplace context.

Our results show no significant direct effect between creative style and innovation behaviors. This study confirms that this relationship might be less obvious than expected. Many factors might be involved in the multifaceted process of innovation, such as, for instance, motivation, attitudes, and support [14]. We add emotion regulation, specifically positive reappraisal and putting into perspective, to this list. Thus, among other factors, a worker’s ability to effectively manage their own emotions might make a difference in whether the generation of novel and original ideas translates into the effective and practical implementation of such ideas. However, as we only focused on positive reappraisal and putting into perspective, future studies might investigate the mediating or moderating role of further emotion-regulation strategies [38,39]. Moreover, an interesting finding of our study is that positive reappraisal displayed the highest association with innovative behaviors. One explanation for this result could be that items such as “I can learn something from the situation” do not assess an emotion-regulation strategy per se but rather a motive for being creative or innovative. Accordingly, creative style may putatively favor the motivation to be innovative, that is, to show innovative behaviors, which in turn translates into innovative behavior itself, in particular when relevant competencies are present, for instance, putting into perspective. In our view, these considerations also pave the way for future studies willing to attempt alternative explanatory models of the variables under study.

### 5.1. Study Limitations

The present study is not without limitations. The cross-sectional nature of the analyzed quantitative data prevents making causality claims, so future research should consider deploying longitudinal designs. Particularly, the results from the rather complex path model, including four variables in total and implying a clear consequential structure, should be considered cautiously due to being based on a short 11-item cross-sectional questionnaire. However, evidence about the reliability and validity of the deployed measures has been provided. Furthermore, the absence of departmental indications is another limitation of the study and does not allow for capturing potential differences between different hospital departments, which are by their nature inhomogeneous. Partly related to this, the analysis of individual-only variables, without consideration of organizational (e.g., climate for innovation [7] or learning climate [40]) and group (e.g., leadership [4,8]) factors, is also a limitation of the study. Future studies may therefore integrate supervisors’ openness to innovations or the organizational learning climate into their investigations. Moreover, the specific sample of Iranian nurses limits the possibility of generalizing the findings beyond healthcare professions in Islamic and Arabic countries, so future research may consider replicating the present study, both identifying wards and considering other countries. However, the measure of innovation behaviors as rated by nurses’ supervisors, even if not immune to bias related to subjective evaluations, could be considered a strength of the study, as it avoids the bias related to self-report assessments. Furthermore, we believe that assessing the validity and reliability of our measures, while considering a low Cronbach alpha for two scales, for physiological reasons (scales consisting of only two items that nevertheless enjoy optimal composite reliability values), gives evidence about the goodness of our results.

### 5.2. Research and Practical Implications

The study results hold important implications both for theory and practice. Regarding research advancements, the full mediation of positive reappraisal in the relationship between creative style and innovation behaviors suggests that this emotion-regulation strategy may be a key explanatory mechanism for how creative style translates into actual innovative behaviors. This finding adds to the growing body of literature on the importance of emotion regulation in creativity and innovation. It highlights the potential utility of interventions that aim to increase positive reappraisal to foster innovation in the workplace. Future research could explore the cognitive mechanisms that underlie the relationship between positive reappraisal and innovation behaviors and examine the generalizability of these findings to other contexts and occupations. In addition, the moderating role of putting into perspective in the relationship between positive reappraisal and innovation behaviors highlights the potential importance of psychological flexibility in facilitating the translation of creative ideas into innovative outcomes. Further research could examine the role of other emotion-regulation strategies in affecting the relationship between creative style and innovation.

In practice, knowledge about anteceding, mediating, and moderating factors may inform the design, development, and implementation of intervention strategies and actions to promote innovation behaviors among nurses. For instance, managers willing to promote innovation in healthcare processes may provide nurses with individual-level training sessions, e.g., mindfulness-based sessions, about emotion regulation, which is known to be a learnable and improvable skill [38,39]. These interventions might ensure a specific focus on positive reappraisal and putting into perspective as key strategies to develop and refine among nurses. Healthcare organizations could then encourage nurses in their wards, through their nursing coordinators, to take work time, when available, to reframe adverse events during their work in a more positive light, as well as to work on their psychological flexibility by seeking diverse experiences and engaging in activities that challenge their preconceived beliefs and assumptions, while adhering to obligatory nursing procedures. As in the case of health promotion interventions among nurses [41], we believe that multilevel strategies, acting on individuals, departments, and entire healthcare facilities, are preferable to single, uncoordinated initiatives. Therefore, interventions promoting innovation among nurses through enhancing emotional regulation skills should enjoy the attention of departmental coordinators and healthcare managers. This may also involve individual nurses eager to make their work more modern and effective.

We also believe that from both theoretical and practical perspectives, scholars and practitioners should focus on differentiating innovation strategies in the work of nurses, who can apply new strategies as much in managing patients as in improving their work experience. Because the strategies may have different theoretical and practical implications, we defer the development of this differential yet more systemic thinking to new studies.

## 6. Conclusions

Due to their front-line knowledge and expertise, nurses are key healthcare professionals that can promote innovation in patient care and hospital management processes and procedures. In the present study, we suggested that creative style and emotion regulation in the form of positive reappraisal and putting into perspective might play a role in facilitating the psychological conditions of innovation behaviors. Hopefully, this knowledge will stimulate managerial actions leading to increased innovation behaviors among nurses to improve the overall quality of healthcare services.

## Figures and Tables

**Table 1:** Characteristics of participants’ demographics (N = 187).

Demographic Characteristics	n	%
Gender		
Women	146	78.9
Men	41	21.1
Age		
Up to 30	42	22.5
31–40	87	46.5
41–50	60	32.2
Over 50	9	4.8
Tenure		
1–10	99	52.9
11–20	61	31.6
21–30	27	14.4
Over 30	2	1.0
Education		
Diploma or below	50	26.7
Bachelor	108	57.8
Master	20	10.7
PhD	9	4.8

**Table 2:** Descriptive statistics, Cronbach’s alphas, and Pearson correlations (N = 187).

	M	SD	CS	PR	PP	IB
Creative style (CS)	3.48	0.84	(0.83)	0.30 **	0.05	0.13
Positive reappraisal (PR)	7.90	1.71		(0.58)	0.56 **	0.48 **
Putting into perspective (PP)	7.91	1.89			(0.55)	0.28 **
Innovation behaviors (IB)	12.91	1.68				(0.80)

Note: Cronbach alphas are reported between brackets. ** *p* < 0.01.

**Table 3:** The indirect effect of creative style on innovation behaviors through positive reappraisal, with the moderator tested at M and ± 1 SD values.

Moderator Value	Point Estimate	BootSE	BootLLCI	BootULCI
Putting into perspective = −1 SD	0.17	0.08	0.05	0.36
Putting into perspective = M	0.25	0.08	0.12	0.45
Putting into perspective = +1 SD	0.33	0.11	0.15	0.58

## Data Availability

Data are available upon request to the corresponding author.
